# Evaluation of the Individual and Combined Toxicity of Fumonisin Mycotoxins in Human Gastric Epithelial Cells

**DOI:** 10.3390/ijms21165917

**Published:** 2020-08-18

**Authors:** Song Yu, Bingxuan Jia, Na Liu, Dianzhen Yu, Aibo Wu

**Affiliations:** SIBS-UGENT-SJTU Joint Laboratory of Mycotoxin Research, CAS Key Laboratory of Nutrition, Metabolism and Food Safety, Shanghai Institute of Nutrition and Health, University of Chinese Academy of Sciences, Chinese Academy of Sciences, Shanghai 200031, China; syu@sibs.ac.cn (S.Y.); jiabingxuan2017@sibs.ac.cn (B.J.); liuna@sibs.ac.cn (N.L.); dzyu@sibs.ac.cn (D.Y.)

**Keywords:** fumonisins, cytotoxicity, ER stress, risk prioritizing, combined toxicity

## Abstract

Fumonisin contaminates food and feed extensively throughout the world, causing chronic and acute toxicity in human and animals. Currently, studies on the toxicology of fumonisins mainly focus on fumonisin B1 (FB1). Considering that FB1, fumonisin B2 (FB2) and fumonisin B3 (FB3) could coexist in food and feed, a study regarding a single toxin, FB1, may not completely reflect the toxicity of fumonisin. The gastrointestinal tract is usually exposed to these dietary toxins. In our study, the human gastric epithelial cell line (GES-1) was used as in vitro model to evaluate the toxicity of fumonisin. Firstly, we found that they could cause a decrease in cell viability, and increase in membrane leakage, cell death and the induction of expression of markers for endoplasmic reticulum (ER) stress. Their toxicity potency rank is FB1 > FB2 >> FB3. The results also showed that the synergistic effect appeared in the combinations of FB1 + FB2 and FB1 + FB3. Nevertheless, the combinations of FB2 + FB3 and FB1 + FB2 + FB3 showed a synergistic effect at low concentration and an antagonistic effect at high concentration. We also found that myriocin (ISP-1) could alleviate the cytotoxicity induced by fumonisin in GES-1 cells. Finally, this study may help to determine or optimize the legal limits and risk assessment method of mycotoxins in food and feed and provide a potential method to block the fumonisin toxicity.

## 1. Introduction

**Figure 1:**
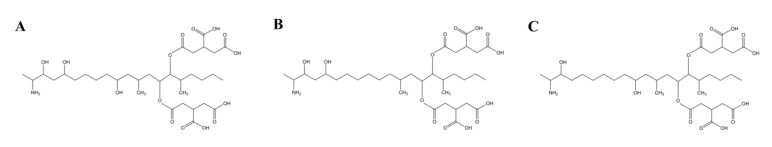
The chemical structure of major fumonisin B: (**A**) fumonisin B1; (**B**) fumonisin B2; and (**C**) fumonisin B3.

As akin natural contaminators produced by *Fusarium*, *Fusarium* mycotoxins are not necessary for fungal growth, while they can fertilize the process of crop infection and cause plant diseases [1,2,3]. According to the Food and Agriculture Organization of the United Nations (FAO), mycotoxin contamination is affecting the entire food chain and causes more than 25% cereal crops loss each year [4]. Fumonisin is a highly toxic low molecular weight *fusarium* mycotoxin, which is produced by a number of *fusarium* species, predominantly *Fusarium verticillioides* and *Fusarium proliferatum* [5,6]. The chemical structure of 28 kinds of fumonisins have been identified by now, which can be divided into A, B, C and P. Among them, fumonisin B1 (FB1), fumonisin B2 (FB2) and fumonisin B3 (FB3) are the main forms. Their structure is characterized by 20-carbon aminopolyhydroxy alkyl chain esterified with two molecules of tricarboxylic acid (-TCA), but they have different amounts of hydroxyl group (-OH) and the positions of the hydroxyl group (-OH) are different as well (Figure 1) [7]. They have often been examined in food and feed all over the world, especially in corn and the products made of corn-flavored ingredients [8,9]. In 2001, a survey of mycotoxin contamination on 4327 grain samples worldwide showed that for major fumonisin B (FBs) positive rates were approximately 27%, 51%, 58%, 56%, and 55%, respectively, in North America, Central Europe, Africa, South Asia, and Southeast Asia. The prevalence in South America was the highest, with a positive rate of 76% (average level of 1.50 mg/kg) [10]. The content of FB contaminators in compound feed and feed ingredients was 93.33% and 83.33%. Most poultry broiler (early) feeds were seriously contaminated by FBs, and the highest detected level in one feed being 12.82 mg/kg in Korea in 2012 [11]. In 2014, the incidence of FB1, FB2 and FB3 in corn products (corn flakes, corn meal and grits) in Shandong province was 98.10%, and the highest levels were 5046, 1350 and 712.10 μg/kg, respectively [12]. Because of the characteristics of this toxin such as its thermal stability and corrosion resistance, they are difficult to eliminate [13]. Once they are absorbed by humans and animals through the food chain, they will threaten human and animal health and cause serious economic losses [14].

Fumonisin B1 (FB1) is the most prevalent member of fumonisin and causes diverse toxic effects in humans and domestic animals, including neurotoxicity, hepatotoxicity and carcinogenesis, resulting from oxidative stress, apoptosis, necrosis and alterations in cell proliferation and differentiation [15,16,17,18,19]. Ceramide synthase catalyzes the formation of ceramide from sphinganine and acyl-CoA as substrates. Several lines of evidence indicate that the toxicity of FB1 caused by the aminopentol backbone competes against the binding of the sphingoid base substrate, whereas the tricarballylic acids interfere with the binding of the fatty acyl-CoA, the accumulation of free sphingoid bases via the inhibition of ceramide synthase leads to the disruption of the sphingolipid metabolism. FB1 can be converted to N-acylation FB1 by ceramide synthase [20,21,22,23]. However, there are limited studies on the toxicity of FB2 and FB3, and no studies have assessed the toxicity of fumonisin B (FBs) mixtures. It is worth mentioning that the current safety limit standards and regulatory formulations of mycotoxins mainly use the toxicology data as references. With the development of toxicology technology, only the consideration of toxicity from one single mycotoxin but not the combined effects (synergistic, additive, and antagonistic) of the mycotoxin mixtures did not obviously reflect the real toxicity of mycotoxins [24,25,26]. However, the data focused on the combination toxicity of mycotoxin are limited. Many investigative studies have shown that the presence of fumonisin family mycotoxins in food and feed is always spatiotemporally synchronized [27,28,29]. Therefore, the evaluation of the toxicological effects of mycotoxin interactions is important for accurately calculating the health risks of such mycotoxin contaminations and formulating relevant safety regulations.

The gastrointestinal tract is often an important target for first exposure to these dietary toxins [30,31]. It is reported that FB1 can affect the health of the gastrointestinal tract (GIT) in animals [32,33]. Nevertheless, the effect of fumonisin on the human gastrointestinal tract was poorly understood. In order to obey the rules of 3R, the human gastric epithelial cell line (GES-1) was used in this study as an in vitro model to evaluate the toxicity of FBs, a class of common fumonisins. Our previous research indicated that the cytotoxicity induced by FB1 was not obvious at 24 h, while all groups (2.5–40 µM) could significantly induce cytotoxicity at 48 h [10]. Therefore, this experiment chose the timepoint of 48 h as the exposure time. First, we assessed the effects of FBs on cell viability, membrane leakage, cell death and the expression levels of endoplasmic reticulum (ER) stress markers to evaluate the rank of the toxicity risk. Furthermore, we assessed the combined toxicity of the fumonisin mixtures and the remission effect of myriocin (ISP-1). These data provide a new reference for establishing and optimizing the law and regulation regarding the safety of mycotoxins and a novel channel to block FBs toxicity.

## 2. Results

### 2.1. FBs Inhibited Cell Proliferation and Increased Lactic Dehydrogenase Levels in GES-1 Cells

**Figure 2:**
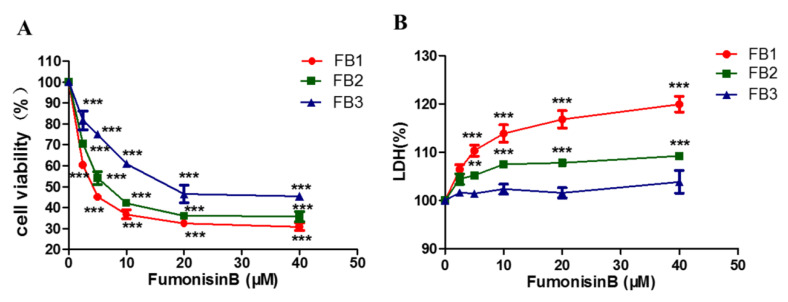
The cytotoxicity of fumonisins B (FBs) on the human gastric epithelial cell line (GES-1) was shown to decrease the cell viability rate and increase the LDH leakage rate. (**A**) Cell viability was assessed by the cell count kit-8 cell proliferation assay. (**B**) Cell membrane integrity was determined by detecting the LDH leakage from the cell media using a cytotoxicity LDH detection kit. These data represented the mean ± SEM of the three individual experiments (** *p* < 0.01, *** *p* < 0.001, analysis of variance (ANOVA) test).

Cell viability and cell membrane leakage are two independent indicators for evaluating cytotoxicity. We used Cell Counting Kit-8 (CCK-8) (DOJINDO) and Cytotoxicity Lactic Dehydrogenase (LDH) assay (DOJINDO) to detect the cytotoxicity of FBs in the human gastric epithelial cell line (GES-1) (Beijing Beina Chuanglian Biotechnology Institute). As shown in Figure 2, the cell viability rate decreased and LDH increased in a dose-dependent manner in the presence of 2.5–40 µM FB1. After 48 h of exposure to FBs, the GES-1 cells had the lowest viability level under 40 μM FBs. The inhibition rates of 40 µM FB1, FB2 and FB3 were 69.16%, 64.32% and 54.60%, respectively (Figure 2A). Simultaneously, when the GES-1 cells were treated with 5 µM FB1 or FB2 for 48 h, the LDH leakage rates were significantly different from the rate in the control group; however, FB3 did not cause a significant increase in LDH. The LDH leakage rate reached the highest level, compared with the control group at a 40 μM dose after 48 h (Figure 2B). The cytotoxicity of FB1 and FB2 was significantly higher than that of FB3.

### 2.2. FBs Induced Cell Death in GES-1 Cells

**Figure 3:**
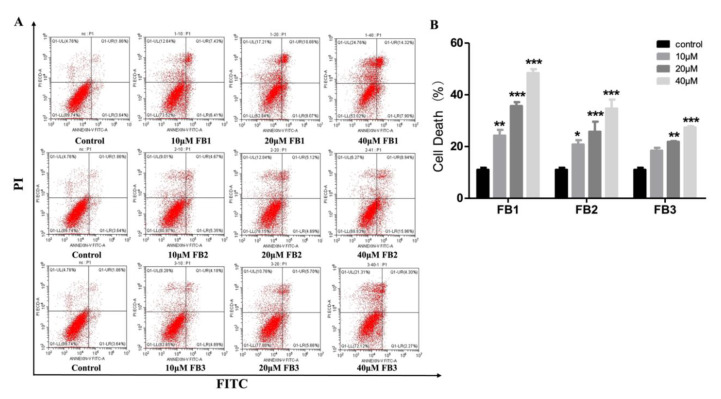
FB1-induced cell death in GES-1 cells. (**A**) Dead cells were marked with Annexin V-FITC/PI dye. The GES-1 cell death rate was analyzed by flow cytometry. (**B**) These data represented the mean ± SEM of the three individual experiments (* *p* < 0.05, ** *p* < 0.01, *** *p* < 0.001, analysis of variance (ANOVA) test).

We further investigated the effect of FBs on GES-1 cell death. Dead cells could be marked with Annexin V-fluoresceine isothiocyanate/propidium iodide (Annexin V-FITC/PI) dye. Flow cytometry analysis was used to quantify the cell death rate in the total cell population. After incubation with FBs (10, 20 and 40 µM) for 48 h, the cell death rates were obviously increased in the FBs treatment groups, compared with the control group (Figure 3A,B). The main form of cell death was necrosis. The highest mortalities were 48.44%, 34.66% and 27.37% in the FB1, FB2 and FB3 group, respectively. FB1 was more lethal than FB2 and FB3.

### 2.3. FBs Induced ER Stress in GES-1 Cells

**Figure 4:**
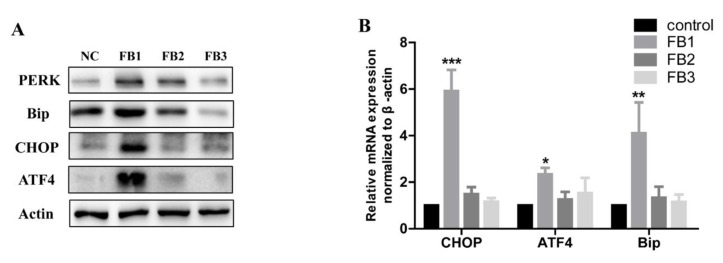
FBs induced ER stress in the GES-1 cells. The levels of ER stress markers were determined by immunoblotting (**A**) and real-time PCR (**B**) after treatment with 20 μM FBs for 48 h. The data represented the mean ± SEM of the three individual experiments (* *p* < 0.05, ** *p* < 0.01, *** *p* < 0.001, analysis of variance (ANOVA) test).

It is well known that the endoplasmic reticulum (ER) stress is activated and induces cell death when the cells are threatened by the external environment [34]. Our previous study has shown that FB1 can induce ER stress in GES-1 cells [10]. We verified whether FB2 and FB3 caused ER stress in GES-1 cells. ER stress biomarkers, protein kinase R-like ER kinase (PERK), glucose regulatory protein 78 (Bip), activated transcription factor 4 (ATF4) and C/EBP homologous protein (CHOP), were detected. The immunoblotting results showed that PERK, Bip, ATF4, and CHOP levels were significantly increased after the treatment with 20 μM FB1 for 48 h (Figure 4A). In addition, the mRNA expression level was also significantly up-regulated (Figure 4B). These results indicated that FB1 can induce ER stress in GES-1 cells significantly. For FB2 and FB3, the expression levels of ER stress markers were also increased. However, they had lower capability. The toxicity order was FB1 > FB2 >> FB3.

### 2.4. Combined Toxicity of FB1, FB2 and FB3 in GES-1 Cells

**Figure 5:**
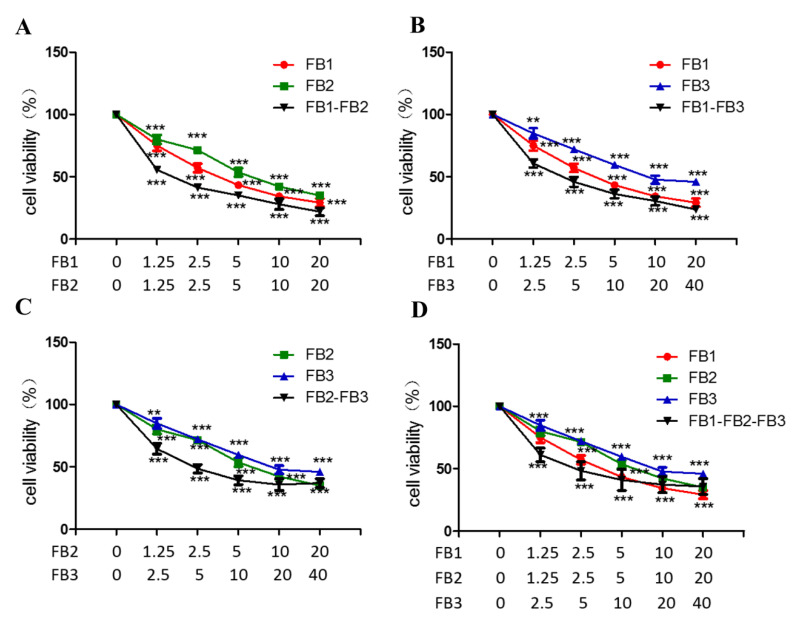
Interactive effects of FB1, FB2 and FB3 on the GES-1 cell viability. (**A**–**D**) GES-1 cells were treated with fumonisin alone or their mixtures for 48 h. The data represented the mean ± SEM of the three individual experiments (** *p* < 0.01, *** *p* < 0.001, analysis of variance (ANOVA) test).

The cell viability of the dose–effect relationship curve for the toxicity of the tested mixture was shown in Figure 5. In the fumonisin B combination, the concentrations shown on the abscissa are: FB1(1.25–20 μM), FB2 (1.25–20 μM) and FB3 (2.5–40 μM). The results showed that the cell viability of the binary combinations remarkedly decreased. The cell viability decreased in a dose-dependent manner in the binary combinations (FB1 + FB2, FB1 + FB3). In particular, the binary combination (20 μM FB1 + 20 μM FB2) were effective in reducing the cell viability compared to other mycotoxin mixtures. However, in the binary and tertiary combinations (FB2 + FB3, FB1 + FB3 + FB3), there was an upward trend in the cell activity at high concentrations, which also suggested antagonistic effects at high concentrations. At a high concentration of 20 μM FB1 + 20 μM FB2 + 40 μM FB3, the cell survival rate decreased to approximately 64.32%.

Table 1 presented the results of the dose–response relationship parameters obtained from in vitro cell viability studies. The correlation coefficient (r) was obtained from the median-effect diagram. This showed a linear correlation coefficient, which meant it was eligible and acceptable for further data analysis using the effect equation. The results showed that the half inhibitory concentration (IC50) value was 1.59 ~ 22.43 μM in binary and tertiary combinations by the isobologram method.

The interaction effect of toxin mixtures mainly include three main different effects: synergistic, additive, and antagonistic. The types of interactions were detemined by the combination index (CI) values that were calculated by the isobologram method [35,36]. The CI of different cytotoxicity levels (IC10–IC75) was shown in Table 2. The results showed that the mixtures of FB1 + FB2 and FB1 + FB3 almost produced synergistic cytotoxicity against GES-1 cells at any level (IC10–IC75). In addition, at the IC10–IC50 level, the FB2 + FB3 combined exposure also had a synergistic effect. At the low cell inhibition level (IC10–IC25), the FB1 + FB2 + FB3 combination produced a synergistic effect and an antagonistic effect at the high cytotoxicity level.

### 2.5. Disruption of Sphingolipid Metabolism Contributes to FBs Cytotoxicity

**Figure 6:**
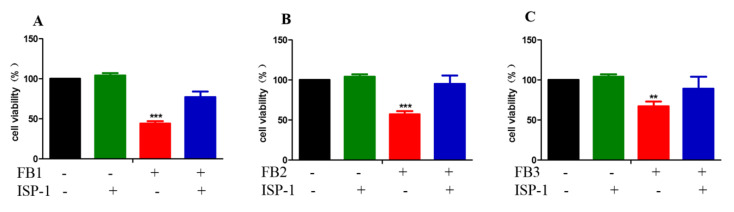
Myriocin (ISP-1) alleviated FB-induced GES-1 cytotoxicity. (**A**) Effect of ISP-1 on FB1-induced cytotoxicity. (**B**) Effect of ISP-1 on FB2-induced cytotoxicity. (**C**) Effect of ISP-1 on FB3 induced cytotoxicity. The data represented the mean ± SEM of the three individual experiments (** *p* < 0.01, *** *p* < 0.001, analysis of variance (ANOVA) test).

Our previous studies have shown that the disruption of sphingolipid metabolism by the inhibition of the ceramide synthase leading to the accumulation of intracellular free sphingoid bases plays an important role in FB1-induced GES-1 cytotoxicity [10]. We then examined whether the disruption of a sphingolipid metabolism also plays an important role in the GES-1 cytotoxicity induced by FB2 and FB3. Myriocin (ISP-1) was an effective specific inhibitor of serine palmityltransferase (SPT), the first enzyme in the sphingomyelin biosynthesis pathway, which prevents the accumulation of sphingoid bases [37]. We measured the cell viability in the presence of myriocin (ISP-1) to evaluate the role of sphingolipid metabolism disorder in FB-induced cytotoxicity. As shown in Figure 6, ISP-1 alleviated the cytotoxicity induced by FBs to varying degrees. This suggested that abnormalities in the sphingolipid metabolism play a key role in the cytotoxicity induced by FB family mycotoxins.

## 3. Discussion

Fumonisins often co-occur in food and feed, in different degrees all over the world. However, toxicological studies often focused on FB1, and there are a few studies on FB2 and FB3. FB1 could cause equine leucoencephalomalacia, pulmonary edema in pig, liver and kidney tumors in rodents, and be associated with esophageal cancer and neural tube defect in humans [15,19,20,23,38]. Nowadays, the formulation of food safety regulations and limit standards about fumonisin only referred to the toxicity data of FB1. This means we ignored the interaction between fumonisins, which can lead to inaccurate safety risk assessments [39]. The gastrointestinal tract, as the first exposure and accumulation location of toxins, is often vulnerable to mycotoxins [40]. Numerous studies have shown that mycotoxins can induce gastrointestinal injury, and FB1 can inhibit the proliferation of Caco-2 and pig intestinal epithelial cells (PIEC) cells [41,42,43,44]. Due to the increasingly restricted regulations on using animals and ensuring their welfare, cell-based systems are more favorable and applicable for toxicity evaluations. The GES-1 cells were used as an in vitro model to evaluate the risk ranking and combination toxicity of FBs in our study.

In order to provide a reliable basis for the investigation of the cytotoxic effects of toxin mixtures, it is first necessary to assess the individual effects of toxins [45]. Initially, we evaluated the effects of FBs on GES-1 cells in many different ways. These were important indexes to evaluate the toxicity of fumonisin in many studies [10,22,46]. Our data showed that FBs could markedly inhibit cell proliferation and increase cell membrane permeability, cell mortality, and the expression level of ER stress markers in varying degrees. The main form of cell death was necrosis. The reason may be that fumonisin, as an exogenous chemical harmful substance, causes pathological damage to GES-1 cells, leading to necrosis, which also indicates that the toxicity induced by fumonisin is irreversible. FB1-induced changes in all aspects were significantly higher than FB2 and FB3, and FB2 was also greater than FB3. Their security risk ranking was FB1 > FB2 >> FB3. FB1, FB2 and FB3 often contaminate both food and feed. Nevertheless, studies on the combined toxicity of fumonisin mixtures are lacking. This study is the first one focused on the combination toxicity of fumonisin. The combination of FB1 + FB2 and FB1 + FB3 almost produced synergistic cytotoxicity against GES-1 cells in all groups. However, the amalgam of FB2 + FB3 and three fumonisins have a synergistic effect at a low dose and an antagonistic effect at a high dose. Similar phenomena have been found in previous studies. Yang et al. found that when deoxynivalenol (DON) family mycotoxins were mixed, their interactions showed a synergistic effect at low concentration and an antagonistic effect at a high concentration [24]. A similar result was shown in v79 cells treated with citrinin and ochratoxin A mixture [47]. Although the respective toxicity of FB2 and FB3 was relatively weak, the toxicity of FB1 would be significantly affected when they were mixed with FB1. The reason may be that the cells stimulated by multiple toxins at the same time change the process of absorption, degradation, accumulation and metabolism of the original single toxin [48,49]. If the interaction of mycotoxin mixtures were not considered in the formulation of safety regulations and limit standards, its safety risk will be underestimated or overestimated, and both could result in a serious health hazard and a lot of economic losses [50,51]. This is a new viewpoint to consider the synergistic and antagonistic effects of fumonisin mixtures in future risk assessment.

Because FB1 has a similar structure to sphingosine, it can impede sphingolipid metabolism by inhibiting ceramide synthase, leading to the accumulation of free sphingoid bases in cells. This accumulation is thought to play a key role in the toxicity of FB1 [52]. Our previous study has shown that the disruption of sphingolipid metabolism is important for FB1-induced GES-1 cytotoxicity. Since the aminopentol backbone and tricarboxylic acid group (-TCA) were present in all FB structures, some reports have shown that FB2 and FB3 can also inhibit ceramide synthase in mice liver [53]. Therefore, we assessed whether the disruption of sphingolipid metabolism also plays a decisive role in FB2- and FB3-induced GES-1 cytotoxicity. We found that ISP-1 could alleviate the cytotoxicity induced by FBs in GES-1 cell and even enable to recover it to the same level as the control group in the FB2 treated group. This also proved that the disruption of sphingolipid metabolism plays an important role in FB-induced toxicity. A previous study reported that the inhibitory ability of FB2 and FB3 to ceramide synthase was lower than FB1, perhaps it is because of their differences in spatial structure [53], this may provide an explanation for the difference in FB toxicity. In the future, researchers might use ISP-1 to treat FB-induced toxicity. This also suggests that similar toxigenic molecular mechanisms are involved in the same class of toxic substances. When we study an unknown toxin, toxicity can be inferred from those toxins which have similar chemical structure and well known toxicity in the future.

## 4. Materials and Methods

### 4.1. Chemicals

Fumonisin B1 (ab142433), fumonisin B2 (ab142434) were from Abcam (Cambridge, MA, USA). Fumonisin B3 (20434) was from Cayman (Ann Arbor, MA, USA). A Cell Counting Kit-8 (CCK-8) (CK04-3000T) and Cytotoxicity Lactic Dehydrogenase (LDH) assay kit (CK12-100T) were obtained from DOJINDO Laboratories (Kumamoto, Japan). Dulbecco’s modified Eagle medium with high glucose (H-DMEM) (SH30243.01) was purchased from HyClone (South Logan, UT, USA). Penicillin–streptomycin–amphotericin B solution (03-033-1B) and trypsin ethylene diamine tetraacetic acid (EDTA) Solution A (0.25%) (03-050-1B) were purchased from BioInd (Kibbutz Beit, Israel). Annexin V-FITC Apoptosis Detection Kit I (556547) was purchased from BD Pharmingen (San Diego, CA, USA). Myriocin (ISP-1) (476300-5MG) was from Merck/Millipore (Billerica, MA, USA). The fetal bovine serum (10099141C) and TRIzol reagent (15596018) were obtained from Invitrogen (Waltham, MA, USA). PrimeScript™ RT Master Mix (RR036A) and SYBR^®^ Premix Ex Taq™ II (RR820A) were from Takara Biomedical Technology (Beijing, China). The bicinchoninic acid assay kit (20201ES90) was purchased from YEASEN Biotechnology (Shanghai, China). The NC membrane (FFN02) was from Beyotime Biotech (Nantong, China). The primary antibodies used for the immunoblotting analyses against Bip (3177S), ATF4 (11815S), PERK (5683S), CHOP (2895S), Actin (3700S) and the secondary antibody IgG labeled with horseradish peroxidase (7076S and 7074S) were purchased from Cell Signalling Technology (Danvers, MA, USA).

### 4.2. Cell Culture and Treatments

The human gastric epithelial cell line (GES-1) was obtained from Beijing Beina Chuanglian Biotechnology Institute (Beijing, China). The GES-1 cells were cultured in H-DMEM (HyClone) with 10% fetal bovine serum (Invitrogen) and penicillin–streptomycin–amphotericin B (BioInd). When the cells were 60–70%, the cells were treated with FBs and/or other agents.

### 4.3. Cell viability Assay and Membrane Leakage Assay

Ten thousand cells were added to each well of the 96-well plate. After 24 h, the culture medium was changed to a 100 μL medium with or without fumonisin. Further training after 48 h, cell viability and membrane leakage were detected. Cell viability and membrane leakage were measured according to the manufacturer’s instructions using a Cell Counting Kit-8 (DOJINDO) and a Cytotoxicity LDH assay kit (DOJINDO), respectively. The results were representative of three independent experiments. Absorbance was recorded with Tecan GENios Pro tablet reader (Tecan).

### 4.4. Cell Death Analysis

Cell death was detected by flow cytometry using an Annexin V-FITC Apoptosis Detection Kit I (BD Pharmingen). The GES-1 cells were treated with FBs (0–40 µM) for 48 h, then they were digested 0.25% trypsin (BioInd) and harvested by centrifugation (1000 rpm/min for 5 min). The cells were stained with 5 μL PI and 5 μL Annexin V-FITC (BD Pharmingen) in 100 μL buffer. The cells were examined by Beckman CytoFlex S (Beckman), and the data were analyzed by the machine’s own analysis software.

### 4.5. Immunoblotting

The cells were treated with FBs for 48 h, then washed once with phosphate buffered saline and harvested. The harvested cells were lysed for 30 min and rotated at 13,000 rpm/min for 20 min at 4 °C. The soluble portion was gathered and the protein concentration was determined by a bicinchoninic acid assay (YEASEN Biotechnology). Then, the 15 μL protein samples (the protein content of each sample was the same) and pre-stained molecular weight markers were subjected to 10% or 12.5% sodium dodecyl sulfate–polyacrylamide gel electrophoresis (this gel had 15 lanes) and transferred to NC membranes (Beyotime Biotech) by electroblotting at 4 °C. Subsequently, the membranes were sealed with 10% skimmed milk powder in Tris-buffered saline and Tween 20 for 2 h at room temperature and incubated overnight in specific antibodies (Actin, ATF4, CHOP, Bip and PERK) (Cell Signalling Technology) diluted 1:1000 or 1:2000 at 4 °C. The secondary antibody IgG was labeled with horseradish peroxidase (Cell Signalling Technology), diluted to 1:2000, and incubated with the membrane at room temperature for 2 h. The staining protein was shown by the Tanon-5200Multi electroluminescence detection system (Tanon).

### 4.6. Real-Time PCR

The total RNA of the GES-1 cells was prepared using TRIzol reagent (Invitrogen). The cDNA was synthesized using PrimeScript™RT Master Mix (Takara). Each sample was quantified using SYBR^®^Premix Ex Taq™II (Takara). The primer sequence and additional information were shown in Table S1. The CT value was detected through StepOnePlus™ Real-Time PCR System (Applied Biosystems™). The gene expression level was analyzed by CT comparison. The expression level of each gene was normalized to the expression level of actin by standard curve method.

### 4.7. Statistical Analysis

The data shown in the study represented the mean ± SEM of the three independent experiments. Tukey or post hoc Bonferroni test after the event was used for one-way or two-way ANOVA, and GraphPad Prism 5 (GraphPad Software Inc., San Diego, CA, USA) was used to evaluate the progressive changes among groups. All the probabilities were two-sided, and *p* < 0.05 was considered statistically significant. All the parameters about combined toxicity were calculated through using the Compusyn software package (ComboSyn Inc., Paramus, NJ, USA, http://www.combosyn.com/, 12/11/2018); for instance, the dose of median–effect (Dm), the slope of the median effect curves (m), the coefficient of linear correlation and the combination index (CI). All methods were carried out in accordance with relevant guidelines and regulations.

## 5. Conclusions

In this study, GES-1 cells were used as in vitro model to evaluate the toxicity of FB1, FB2 and FB3. We found that they could significantly reduce the cell viability, increase membrane leakage, cell death and induce ER stress. We confirmed that their toxicity potency was FB1 > FB2 >> FB3. With the widespread study of fumonisin mixtures on GES-1 cells, the interactions amongst them lead to synergistic or antagonistic effects. We also found that myriocin (ISP-1) could alleviate the cytotoxicity induced by FBs in GES-1 cell. These data implied that FBs could cause gastrointestinal damage through inducing sphingolipid metabolism disorder and activating ER stress. Finally, this study helps to determine or optimize the legal limits of mycotoxins in food and feed. Particularly, more attention should be focused on the toxins with synergistic effect at low concentration. We also provide a method to intervene in the FBs toxicity. In the future, we will explore the molecular mechanisms of the synergistic and antagonistic effects between them.

## Figures and Tables

**Table 1:** Dose–effect relationship parameters for cytotoxicity by fumonisins in GES-1 cells.

	Dm	M	r
FB1	4.43919	−0.70722	0.97471
FB2	7.45614	−0.75675	0.98928
FB3	22.43032	−0.69375	0.96569
FB1 + FB2	1.59058	−0.51736	0.99049
FB1 + FB3	4.39135	−0.566	0.98716
FB2 + FB3	5.972	−0.40263	0.95451
FB1 + FB2 + FB3	5.46792	−0.36484	0.94273

Dm, the median-effect dose; m, the slope of median-effect curves; r, the correlation coefficient.

**Table 2:** Combination index (CI) for cytotoxicity by individual fumonisins and their mixtures in GES-1 cells.

Mycotoxin	Combination Ratio	IC10	IC25	IC50	IC75
		Combination Index			
FB1:FB2	1:1	0.17	0.31	0.57	1.05
FB1:FB3	1:2	0.30	0.46	0.69	1.04
FB2:FB3	1:2	0.06	0.20	0.66	2.27
FB1:FB2:FB3	1:1:2	0.06	0.28	1.23	5.41

IC, inhibitory concentration; CI < 1, indicates synergistic effects; CI = 1, indicates additive effects and CI > 1 indicates antagonistic effects.

## Supplementary Materials

Can be found at https://www.mdpi.com/1422-0067/21/16/5917/s1. Table S1. RT-PCR primers for the ER stress genes.

## Abbreviations

FBs	Fumonisin Bs
FB1	Fumonisin B1
FB2	Fumonisin B2
FB3	Fumonisin B3
CCK-8	Cell Counting Kit-8
LDH	lactic dehydrogenase
Annexin V-FITC/PI	Annexin V-fluoresceine isothiocyanate/propidium iodide
ER	Endoplasmic reticulum
EDTA	Ethylene diamine tetraacetic acid
PERK	Protein kinase R-like ER kinase
CHOP	C/EBP homologous protein
Bip	Glucose-regulated protein 78
ATF4	Activating transcription factor 4
GES-1	Human gastric epithelial cell line
H-DMEM	Dulbecco’s modified eagle medium with high glucose
GIT	Gastrointestinal tract
SPT	Serine palmitoyltransferase
PIEC	Pig intestinal epithelial cells
ISP-1	Myriocin
IC	Inhibitory concentration
CI	Combination index
DON	Deoxynivalenol
