# Supplementary material for: Evaluation of the Individual and Combined Toxicity of Fumonisin Mycotoxins in Human Gastric Epithelial Cells
Source: Int J Mol Sci. 2020 Aug 18;21(16):5917. doi: 10.3390/ijms21165917 (PMC7460643; doi:10.3390/ijms21165917)
Supplement: Supplementary file 1 [file ijms-21-05917-s001.pdf]

Supplementary information for

S1 Table. RT-PCR primers for the ER stress genes.

**Gene name**	**Primer**
GRP78	F: TTGTTCTTGTTGGTGGCTCGACTC<br>R: GACAGCAGCACCATACGCTACAG
ATF4	F: GTTCCTGCTGCCTGGTGTCTTG<br>R: GCACGCCACCTTCTCAATTCATTC
CHOP	F: CTGCTTCTCTGGCTTGGCTGAC<br>R: TTGGTCTTCCTCCTCTTCCTCCTG
β-actin	F:CCATCATGAAGTGTGACG<br>R:GCCGATCCACACGGAGTA
